# The Role of CD4^+^CD8^+^ T Cells in HIV Infection With Tuberculosis

**DOI:** 10.3389/fpubh.2022.895179

**Published:** 2022-05-27

**Authors:** Shi Zou, Yuting Tan, Yanni Xiang, Yang Liu, Qi Zhu, Songjie Wu, Wei Guo, Mingqi Luo, Ling Shen, Ke Liang

**Affiliations:** ^1^Department of Infectious Diseases, Zhongnan Hospital of Wuhan University, Wuhan, China; ^2^Wuhan Research Center for Infectious Diseases and Cancer, Chinese Academy of Medical Sciences, Wuhan, China; ^3^Department of Intensive Care Medicine, Yichang Central People's Hospital, Yichang, China; ^4^Zhongnan Hospital of Wuhan University, Wuhan University, Wuhan, China; ^5^School of Economics and Management, Wuhan University, Wuhan, China; ^6^Wuhan Pulmonary Hospital, Wuhan Institute for Tuberculosis Control, Wuhan, China; ^7^Department of Nosocomial Infection Management, Zhongnan Hospital of Wuhan University, Wuhan, China; ^8^Department of Pathology, Zhongnan Hospital of Wuhan University, Wuhan, China; ^9^Department of Pathology, School of Basic Medical Sciences, Wuhan University, Wuhan, China; ^10^Department of Microbiology and Immunology, Center for Primate Biomedical Research, University of Illinois College of Medicine, Chicago, IL, United States; ^11^Hubei Engineering Center for Infectious Disease Prevention, Control and Treatment, Wuhan, China

**Keywords:** HIV, tuberculosis (TB), double positive (DP) T cells, CCR5, granzyme A

## Abstract

**Background:**

Tuberculosis (TB) is an important opportunistic infection in acquired immunodeficiency diseases (AIDS). Although the frequency of CD4^+^CD8^+^ double-positive (DP) T cells has been observed to increase in pathological conditions, their role (phenotypic and functional) is poorly described, especially in human immunodeficiency virus (HIV) infection with TB (HIV/TB (HT) coinfection).

**Methods:**

The percentage and phenotypic and functional properties of peripheral blood DP T cells in patients with HT coinfection in comparison to uninfected controls and to patients with HIV or TB mono-infection were analyzed by direct intracellular cytokine staining (ICS).

**Results:**

Total and CD4^low^CD8^high^ DP T cells were significantly increased in patients with both HIV and TB mono-infection, especially in patients with HT coinfection. Compared with healthy controls (HCs), the percentage of DP T cells expressing chemokine receptor 5 (CCR5) in patients with HT coinfection was significantly higher. Compared with HCs and patients with TB, a lower percentage of tumor necrosis factor α (TNF-α) secreting DP T cells and a higher percentage of granzyme A-secreting DP T cells were observed in patients with HIV mono-infection and HT coinfection, respectively. In addition, DP T cells expressed more cytolytic markers (granzyme A and perforin) than CD4^+^ T cells, but similarly to CD8^+^ T cells in patients with HT coinfection.

**Conclusions:**

Our data suggested that HT coinfection resulted in a marked increase in DP T cells, especially the CD4^low^CD8^high^ subpopulation. DP T cells may be susceptible to HT coinfection, and have the same cytotoxic function as CD8^+^ T cells.

## Introduction

Tuberculosis (TB), caused by *Mycobacterium tuberculosis* (MTB) infection, is the most common opportunistic infection in patients with acquired immunodeficiency diseases (AIDS) (1). Patients with human immunodeficiency virus (HIV) infection and TB represent a serious threat to public health worldwide (2, 3). HIV may potentiate mycobacterial virulence factors and has a significant impact on the progression of TB (4, 5). TB also promotes cellular susceptibility to HIV and drives HIV replication (5, 6).

Human peripheral T cells are a crucial component of the adaptive immune system and commonly express CD4 or CD8 on their surface, with specific helper or cytolytic functions. However, a subpopulation of T cells with coexpression of CD4 and CD8, namely CD4^+^CD8^+^ double-positive (DP) T cells, were found in several studies and accounted for about 1–2% of circulating human T lymphocytes in healthy people (7). According to the expression levels of CD4 and CD8 molecules on DP T cells, three subpopulations have been commonly divided: a subpopulation with high CD4 expression but low CD8 expression (CD4^high^CD8^low^), a subpopulation with high CD8 but low CD4 (CD4^low^CD8^high^) expression, and a less numerous subpopulations of CD4^high^CD8^high^ (8).

An increased frequency of DP T cells has been observed in patients with autoimmune diseases, various types of cancers, and viral infections (9). DP T cells also exhibit cytokine production and cytolytic function (10). In HIV or TB mono-infection, the percentage of DP T cells can increase up to 20% of all circulating lymphocytes (11, 12). As is known, HIV can invade target cells through the CD4 receptor, CC chemokine receptor 5 (CCR5), and/or CXC chemokine receptor 4 (CXCR4). DP T cells not only have these receptors but also have highly active memory phenotypes and are highly enriched in the intestinal tract (13), making such cells sensitive to HIV. Indeed, both *in vivo and in vitro* studies have been shown that DP T cells can be infected with HIV (14, 15). Moreover, evidence suggested that HIV-specific DP T cells exhibited polyfunctionality with the characteristics of CD4^+^ and CD8^+^ T cells, including the production of interferon-gamma (IFN-γ) and interleukin-2 (IL-2) during acute and chronic HIV infection (16). During active MTB infection in cynomolgus macaques, DP T cells had distinct patterns and greater cytokine production in peripheral, airway, and lung granulomas than CD4^+^ or CD8^+^ T cells (12). Therefore, DP T cells may play an important role in immunity against HIV or MTB.

Intracellular cytokine staining (ICS) has been widely used in TB studies. Although stimulation of peripheral blood mononuclear cells (PBMCs) by a specific antigen may reflect cell-specific responses to MTB, it cannot represent the actual immune response of the human body as there is seldom so high concentration of antigens in the blood. Direct ICS (without specific antigen stimulation) was well described to inspect the natural cellular responses in MTB infection, and this method proved to be reliable in describing the immune status of MTB infection in our previous and other studies (17–19). Therefore, in this study, we used direct ICS to explore the function of DP T cells in HIV/TB (HT) coinfection. Comparison of the three pathological states and healthy individuals is more conducive to understanding the function of DP T cells. Hence, the present study aimed to investigate the phenotypic and functional properties of DP, CD4^+^, and CD8^+^ T cells in HIV infection with TB (uninfected controls, patients with HIV or TB as a control group).

## Materials and Methods

### Study Population

Subjects enrolled in the prospective research were divided into four groups (1). *Healthy control (HC) group*: subjects with no history of chronic inflammatory diseases or no signs of infection in at least 2 weeks before peripheral blood collection at the physical examination center of Zhongnan Hospital of Wuhan University were recruited. All were screened for HIV antibodies (Abs), chest x-ray, and interferon gamma release assay (IGRA) to rule out HIV and MTB infection (2). *TB group*: individuals recruited from Wuhan Pulmonary Hospital with a confirmed diagnosis of TB by etiological or histopathological methods (positive smear or culture, and/or positive MTB DNA test, and/or positive Xpert MTB/RIF test, and/or histopathological evidence supporting TB) and negative HIV Abs (3). *HIV group*: individuals recruited with HIV infection confirmed by HIV RNA detection and HIV antibody screening, but excluding MTB infection by chest X-ray and IGRA at the AIDS Clinical Guidance and Training Center, Zhongnan Hospital of Wuhan University (4). *HT group*: individuals recruited with confirmed HIV and TB infection by the above diagnostic method at Zhongnan Hospital of Wuhan University.

### Sample Collection and Isolation of PBMCs

Peripheral blood mononuclear cells were isolated from freshly collected EDTA-coagulated blood using Lymphoprep (Axis-Shield, Norway) with density gradient centrifugation. Cell pellets were treated with 5 ml of RBC lysis buffer (Sigma-Aldrich) for 10 min, followed by washing one time with 5% fetal bovine serum-phosphate buffered saline (FBS–PBS). PBMCs were then counted and cryopreserved until the next step experiments.

### Abs and Reagents

The following Abs were used for short-term culture or surface marker and ICS for flow cytometry (all Abs were from Biolegend): anti-CD3-PerCP-cy5.5, anti-CD8-APC-Cy7, anti-CD4-PE-Cy7, anti-CCR5-PE, anti-CXCR3-APC, anti-CXCR4-PE, anti-CCR7-APC, anti-granzyme A-PE, anti-Perforin-APC, anti-IFN-γ-PE, and anti-TNF-α-APC. The reagents listed below were all commercial products: brefeldin A (GolgiPlug, BD Biosciences), Cytofix/Cytoperm (BD Biosciences), and Perm buffer (BD Biosciences).

### Direct ICS Assay

This procedure was done exactly the same as described previously (19). In brief, PBMCs were incubated for 1 h with medium in the presence of CD28 (1 μg/ml) and CD49d (1 μg/ml) mAbs in a 200 ml final volume in round-bottom 96-well plates at 37°C, 5% CO_2_, followed by a 5-h incubation in the presence of brefeldin A (GolgiPlug; BD Biosciences). At the end of the incubation, cells were washed one time with 2% FBS–PBS and stained at room temperature for at least 15–30 min with surface marker Abs (CD3, CD4, CD8, CCR5, CXCR3, CXCR4, and CCR7). After the next 45 min permeabilization (Cytofix/Cytoperm; BD Biosciences), another 45 min was performed for ICS (granzyme A, perforin, IFN-γ, and tumor necrosis factor α (TNF-α)). Finally, cells were resuspended in 2% formaldehyde and subjected to flow cytometry analysis. To ensure specific immunostaining in direct or indirect ICS, IgGs of matched isotype were served as negative controls.

### Statistical Analysis

Flow cytometric data were analyzed with FlowJo version 7.6.1 for Windows. Statistical analysis of the data was performed with GraphPad Prism version for Windows. Non-parametric data analysis used the Mann–Whitney *U* test for median comparison. Multiple linear regression was employed to identify CD4 count associated with DP frequency. All reported *p*-values were two-tailed, and a *p*-value of 0.05 or less was considered significant.

## Results

### Characteristics of Enrolled Individuals

Totally, 165 individuals (35 in the HC group, 60 in the TB group, 30 in the HIV group, and 40 in the HT group) were enrolled and designated into different groups following the rules described in the methods. The features of these individuals are summarized in Table 1. There were no statistical differences in age and sex composition among all groups. To generally evaluate the immune status of each group, CD4^+^ T lymphocyte count (CD4 count) was performed by flow cytometry. The HC group showed the highest median CD4 count as 889 cells/μl [735, 961], while the TB group caused an obvious loss of CD4^+^ T cells to the number of 701 cells/μl (609, 915), which is between the number of the HIV group (413 cells/μl (328, 596)) and the HC group. The HT group showed the lowest median CD4 count undoubtedly as 81 cells/μl (43, 166). All individuals were tested for lymphocyte phenotypes in PBMC (Table 1), and only 48 individuals were tested for cytokines and chemokine receptors (Table 2).

**Table 1:** Clinical characteristics of study population.

**Characteristic**	**Patient group**				
	**HC (*n* =35)**	**TB (*n* =60)**	**HIV (*n* =30)**	**HIV/TB (*n* =40)**	***P*-value**
Age, median years (range)	32 (28, 37)	36 (29, 56)	39 (30, 47)	40 (33,51)	0.21
Male, no. (%)	17 (49)	35 (58)	19 (63)	26 (65)	0.08
Pulmonary tuberculosis	-	58 (97)	-	35 (87)	
Extrapulmonary tuberculosis	-	2 (3)	-	5 (13)	
Receiving ART, *n*(%)	-	-	6 (20)	7 (18)	
CD4 count (cells/μl), median	889 (735, 961)	701 (609, 915)	413 (328, 596)	81 (43, 166)	0.00

*Data are n (%) or median (interquartile range (IQR)). Data are for participants with human immunodeficiency virus (HIV) and without HIV included in this analysis*.

**Table 2:** Clinical characteristics of individuals tested for cytokines and chemokine receptors.

**Characteristic**	**Patient group**				
	**HC (*n* =10)**	**TB (*n* =14)**	**HIV (*n* =11)**	**HIV/TB (*n* =13)**	***P*-value**
Age, median years (range)	30 (23, 38)	41 (29, 54)	39 (30, 49)	35 (27,45)	0.16
Male, no. (%)	6 (60)	9 (64)	7 (64)	9 (69)	0.20
Pulmonary tuberculosis	-	14 (100)	-	11 (85)	
Extrapulmonary tuberculosis	-	0 (0)	-	2 (15)	
Receiving ART, *n*(%)	-	-	2 (8)	0 (0)	
CD4 count (cells/μl), median	813 (706, 944)	711 (599, 896)	378 (275, 577)	77 (42, 166)	0.00

*Data are n (%) or median (IQR). Data are for participants with HIV and without HIV included in this analysis*.

### The HT Group Has a Higher Proportion of Circulating DP T Cells

**Figure 1:**
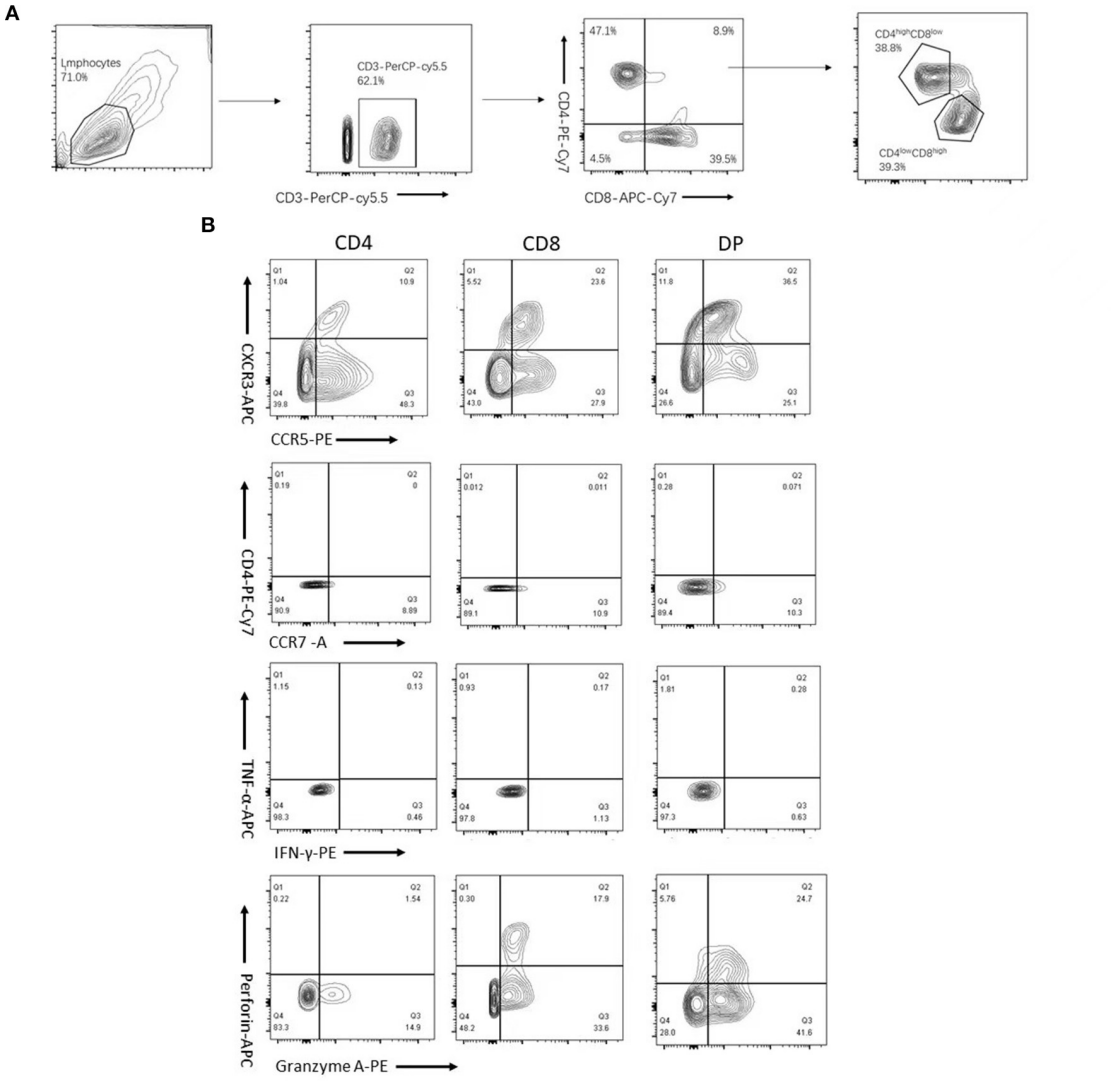
Flow cytometric gating strategy. CD3^+^ subsets of CD4^+^, CD8^+^, and CD4^+^CD8^+^ (double-positive (DP)) T cells were gated as described in Section “Materials and methods.” And DPT cells were further divided into CD4^high^CD8^low^ and CD4^low^CD8^high^ T cells based on the expression levels of CD4 and CD8 molecules **(A)**. Plots depicting CXCR3, chemokine receptor 5 (CCR5), CXC chemokine receptor 4 (CXCR4), CCR7, interferon-gamma (IFN-γ), tumor necrosis factor α (TNF-α), perforin, and granzyme A expression or production of gated CD4^+^, CD8^+^, and DP T cells **(B)** are shown.

**Figure 2:**
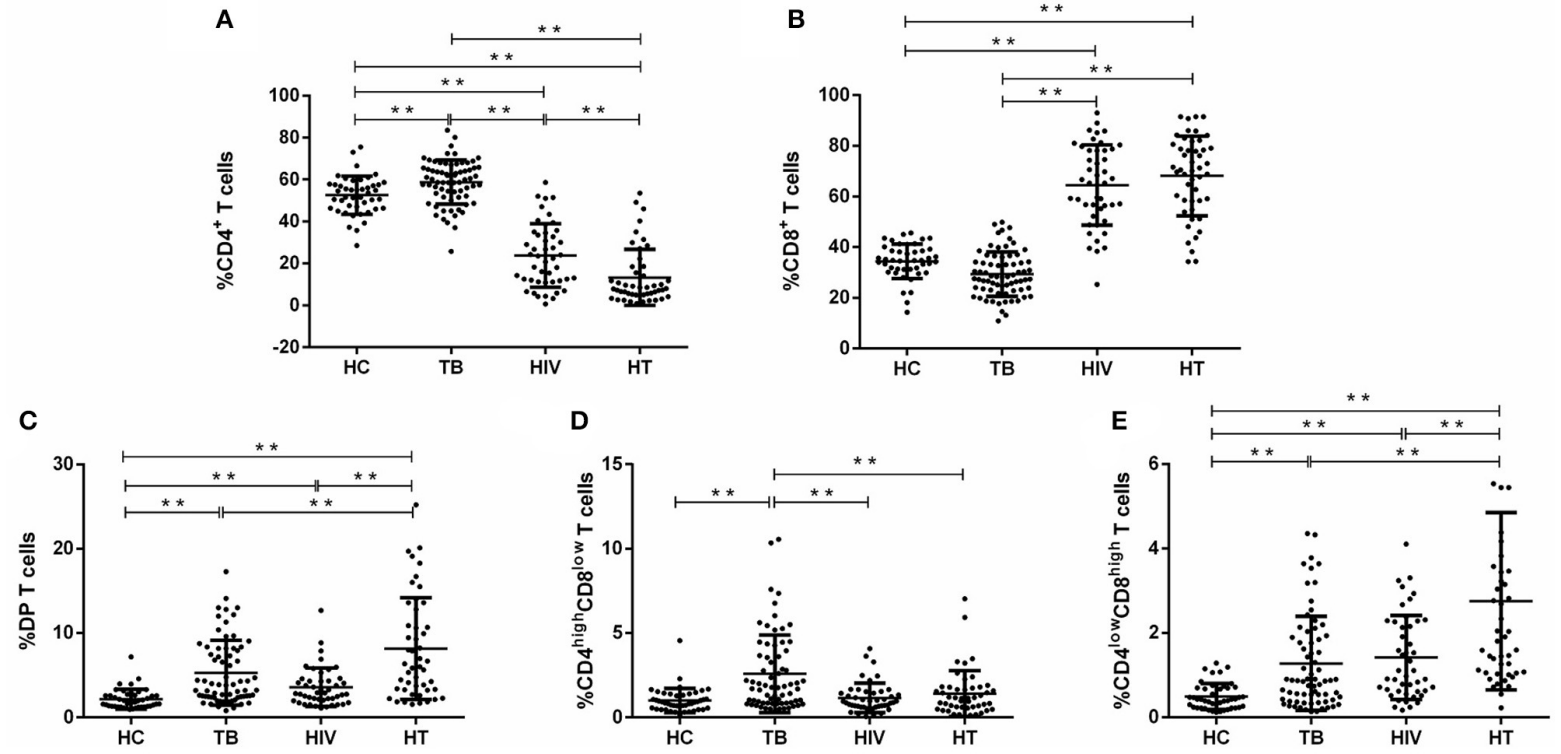
Proportions of CD3^+^ T-lymphocyte subsets among each group. The percentage of CD4^+^, CD8^+^, and CD4^+^CD8^+^ (DP), [**(A–C)**, respectively], and CD4^high^CD8^low^ and CD4^low^CD8^high^ subsets of DP T cells [**(D,E)**, respectively] among gated CD3^+^ lymphocytes are shown. *P*-value calculated using the Mann–Whitney *U* test. Statistically significant differences between the groups are indicated as follows: *p < 0.05 and **p < 0.01.

To characterize the dynamics in DP T cells during the course of HT coinfection, the percentages of CD4^+^, CD8^+^, total DP, CD4^high^CD8^low^, and CD4^low^CD8^high^ T cells in CD3^+^ lymphocytes were examined in the HC, TB, HIV, and HT group. The flow cytometric gating strategy is illustrated in Figure 1. As shown in Figures 2A,B, compared with the HC group, the TB group had a significantly higher fraction of CD4^+^ T cells and a similar fraction of CD8^+^ T cells. The HIV group had a significantly lower fraction of CD4^+^ T cells and a higher percentage of CD8^+^ T cells compared with the HC group (Figures 2A,B). Similar to the HIV group, the HT group also had a significantly lower fraction of CD4^+^ T cells and a higher percentage of CD8^+^ T cells than the HC and TB group. The percentage of CD4^+^ T cells was lower in the HT group than in the HIV group (Figures 2A,B). A much higher percentage of DP T cells was observed in the TB, HIV, and HT group compared with the HC group (Figure 2C). The percentage of DP T cells was even higher in the HT group than in the TB and HIV group (Figure 2C). Although there was a significant difference in CD4 count between the HIV and HT groups, no significant correlation was observed between CD4 count and DP frequency in the HIV (Spearman's ρ = 0.16, *p* = 0.54) and HT group (Spearman's ρ = 0.14, *p* = 0.4) (Supplementary Figure S1).

Then, DP T cells were further divided into CD4^high^CD8^low^ and CD4^low^CD8^high^ T cell subsets. It was shown that the percentage of CD4^high^CD8^low^ and CD4^low^CD8^high^ T cells was significantly higher in the TB group than in the HC group (Figures 2D,E). The HIV and HT group had a significantly higher fraction of CD4^low^CD8^high^ T cells and a fraction of CD4^high^CD8^low^ similar to the levels of the HC group (Figures 2D,E). It is worth noting that the percentage of CD4^low^CD8^high^ T cells was even higher in the HT group than in the TB and HIV group (Figures 2D,E).

### Higher Proportion of DP T Cells Expressing CCR5 in the HT Group

**Figure 3:**
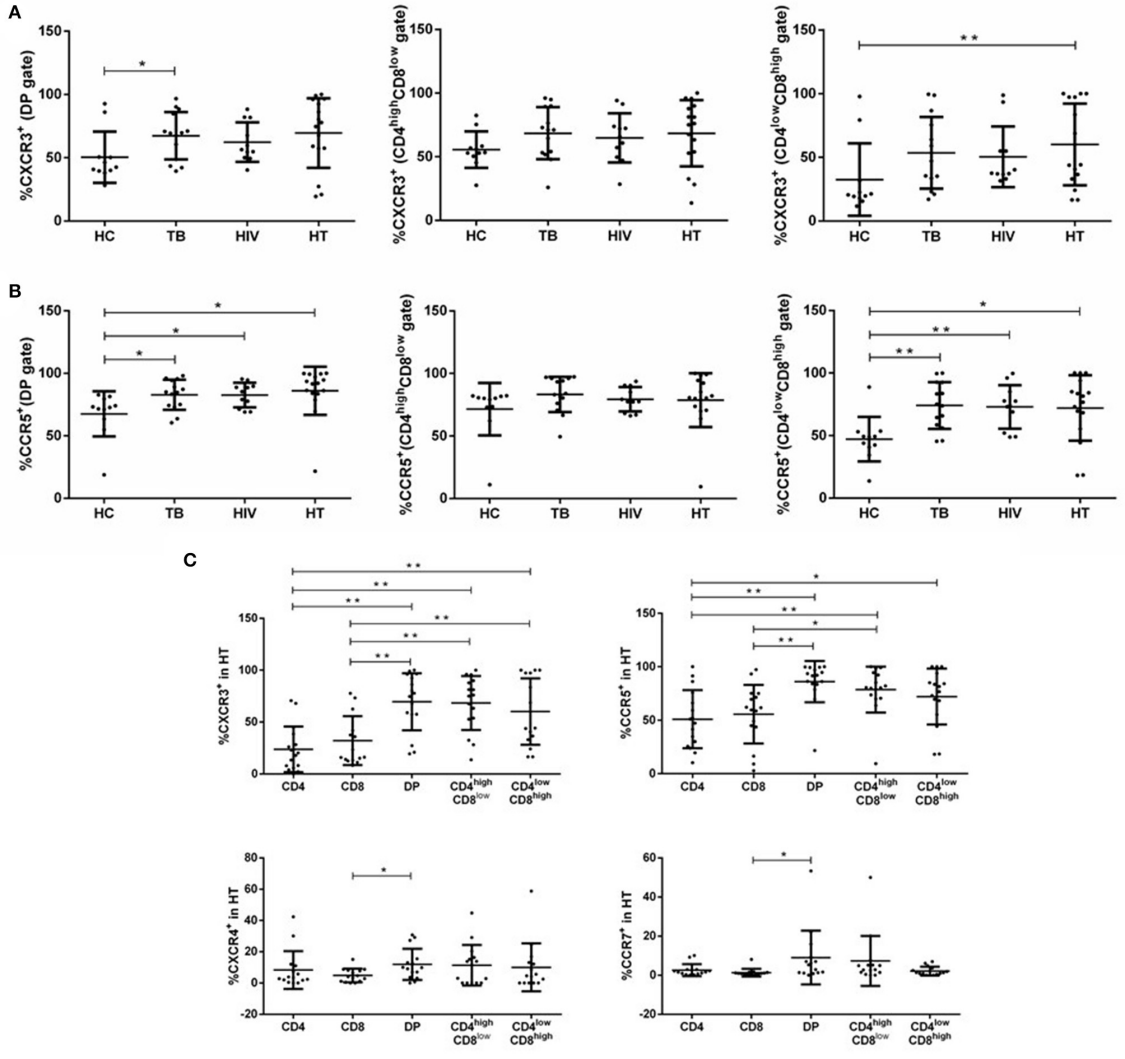
Distribution of chemokine receptors among subsets of DP T cells in each group. Percentages of DP T subsets expressing CXCR3 **(A)** and CCR5 **(B)** within each group are shown. Subjects for chemokine receptor expression of CXCR3, CCR5, CXCR4, and CCR7 in T-lymphocyte subsets in the HT group are shown in **(C)**. *P*-value calculated using the Mann–Whitney *U* test. Statistically significant differences between the groups are indicated as follows: *p < 0.05 and **p < 0.01.

Different HIV-1 variants use either CCR5, CXCR4, or both. Chemokine receptors, such as CXCR3, CXCR4, CCR5, and CCR7, play a critical role in many infectious diseases, including AIDS and TB (20, 21). In our study, the expression of chemokine receptors (CXCR3, CXCR4, CCR5, and CCR7) of DP T cells in each group was examined. Compared with the HC group, DP T cells expressed significantly greater CXCR3 and CCR5 in the TB group (Figures 3A,B). CD4^low^CD8^high^ T cells expressed only greater CCR5 in the TB group compared with the HC group (Figure 3B). Compared with the HC group, total and CD4^low^CD8^high^ DP T cells expressed higher CCR5 in the HIV group (Figure 3B). Compared with the HC group, the proportion of DP expressing CCR5 and the proportion of CD4^low^CD8^high^ T cells expressing CXCR3 and CCR5 were greater in the HT group (Figures 3A,B). No differences in the percentage of total, CD4^high^CD8^low^, or CD4^low^CD8^high^ DP T cells expressing CXCR4 or CCR7 were observed among all groups (Supplementary Figures S2A,B).

To distinguish DP T cells from CD4^+^ or CD8^+^ T cells, we further compared the percentage of each cell type expressing chemokine receptors in the HT group. DP T cells expressed higher CXCR3 and CCR5 than CD4^+^ and CD8^+^ T cells, and significantly more CXCR4 and CCR7 than CD8^+^ T cells, but not CD4^+^ T cells (Figure 3C). The proportion of CD4^high^CD8^low^ cells expressing CXCR3 and CCR5 was > CD4^+^ and CD8^+^ T cells (Figure 3C). CD4^low^CD8^high^ T cells expressed more CXCR3 compared with both CD4^+^ and CD8^+^ T cells and greater CCR5 than CD4^+^ T cells, but not CD8^+^ T cells (Figure 3C).

### DP T Cells Producing Higher Granzyme a and Lower TNF-α in the HT Group

**Figure 4:**
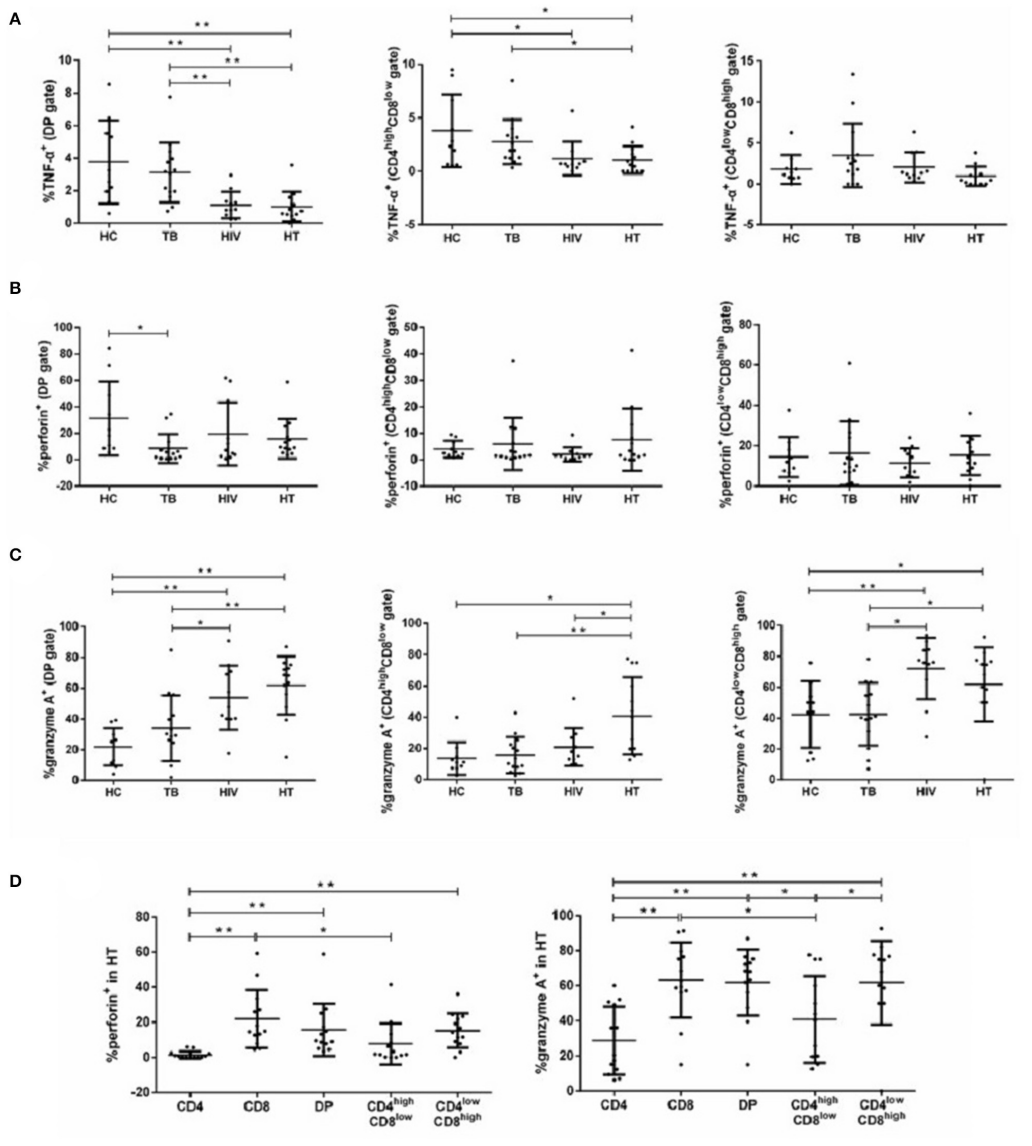
Function of DP T cells among each group. Percentages of DP T subsets producing TNF-α **(A)**, perforin **(B)**, and granzyme A **(C)** in each group are shown. Subjects that produce cytokines of perforin and granzyme A in T-lymphocyte subsets among the HT group are shown in **(D)**. *P*-value calculated using the Mann–Whitney *U* test. Statistically significant differences between the groups are indicated as follows: *p < 0.05 and **p < 0.01.

Comparisons of proinflammatory cytokines (IFN-γ, TNF-α, perforin, and granzyme A) among patients with HIV and/or TB infection are shown in Figure 4. As shown in Figure 4B, the TB group had significantly lower percentages of DP T cells producing perforin than the HC group. Compared with the HC group, DP and CD4^high^CD8^low^ T cells produced less TNF-α in the HIV group (Figure 4A). In contrast, total and CD4^low^CD8^high^ DP T cells produced more granzyme A in the HIV group than in the HC group (Figure 4C). Similar to the HIV group, the percentage of TNF-α-producing total and CD4^high^CD8^low^ DP T cells was significantly lower in the HT group than in the TB and HC group (Figure 4A). The percentage of granzyme A-producing total and CD4^low^CD8^high^ DP T cells was significantly higher in the HT group than in the TB and HC group (Figure 4C). In addition, granzyme A-producing CD4^high^CD8^low^ T cells were significantly higher in the HT group than in the TB, HIV, and HC group (Figure 4C). No differences in the percentage of IFN-γ-producing total, CD4^high^CD8^low^, or CD4^low^CD8^high^ DP T cells were observed among all groups (Supplementary Figure S3A).

We also compared the percentage of each cell type producing proinflammatory cytokines in the HT group. Total and CD4^low^CD8^high^ DP T cells produced higher perforin and granzyme A than CD4^+^ T cells, but not CD8^+^ T cells. CD4^high^CD8^low^ T cells produced lower perforin and granzyme A compared with CD8^+^ T cells, but not with CD4^+^ T cells. No differences in the percentage of total, CD4^high^CD8^low^, or CD4^low^CD8^high^ DP T cells producing IFN-γ and TNF-α were observed in the HT group (Supplementary Figure S3B).

## Discussion

Several studies have reported an increased percentage of peripheral DP T cells under pathological conditions (8, 11). However, this is the first study to characterize the phenotype and functions of DP T cells and distinguish them from CD4^+^ and CD8^+^ T cells during HT coinfection. Our results showed that DP T cells were significantly increased in the TB and HIV group, especially during HT coinfection. It is worth noting that the highest percentages of DP T cells in the HT group were mainly attributed to an increase in the CD4^low^CD8^high^ subpopulation, and an increase in the CD4^low^CD8^high^ subpopulation was also observed in the HIV group, while the TB group displayed mostly CD4^low^CD8^high^ and CD4^high^CD8^low^ DP T cells. CD4^low^CD8^high^ DP T cells are CD8αβ, which are generated from highly purified CD8^+^ T cells (9). *In vivo* and *in vitro* studies suggested that peripheral CD4^high^CD8^low^ T cells might be derived from CD4^+^T cells and represent a subset of late differentiated effector CD4^+^ T cells, while CD4^low^CD8^high^ T cells might be derived from CD8^+^T cells activated during viral infection (10, 11). Consistently, the findings of a previous study showed that CD8^+^ T cells from patients mono-infected with HIV were capable of upregulating CD4 on CD8^+^ T cells (22). Given that during post-HIV infection CD4 may be downregulated on the surface of CD4^high^CD8^low^ T cells or CD4^high^CD8^low^ T cells may be more vulnerable to HIV as CD4^+^ T cells, these may explain our data that this subpopulation increased only in the periphery of the TB group but not in the HIV and HT group. Similarly, we found that TB coexistence had a higher percentage of CD4^low^CD8^high^ T cells than mono-HIV infection, which may be related to TB accelerating the attack of HIV on CD4^+^ T cells and promoting the downregulation of surface CD4 of CD4^+^ T cells. Moreover, HIV infection and TB did not yield the same patterns of DP T cells, suggesting the existence of antigen specificities.

Chemokine receptors play a critical role in many infectious diseases. To infect a target cell, the HIV envelope glycoprotein gp120 has to interact with the cellular receptor CD4 and the co-receptor, chemokine receptors CC or CXC (20). Chemokine receptors are also essential for granuloma development in TB (21). In normal human blood, CXCR4 is primarily expressed on naive lymphocytes, whereas CCR5 expression is primarily limited to memory lymphocytes (23). It is well-established that, in addition to the CD4 molecule, HIV requires one or more chemokine receptors (especially CXCR4 or CCR5) as co-receptors for attachment, fusion, and entry into host cells (24). Our results showed that total and CD4^low^CD8^high^ DP T cells expressing CCR5 in the TB, HIV, and HT group were significantly higher than those in the HC group. However, we also found that there was no significant difference of DP and CD4^low^CD8^high^ T cells expressing CCR5 between the disease groups (TB,HIV, and HT group), suggesting coexistence with HIV or coexistence with TB may not influence CCR5 expression on circulating DP and CD4^low^CD8^high^ T cells in comparison to TB or HIV mono-infection. Given that CD4^low^CD8^high^ T cells may be derived from activated CD8^+^ T cells, our results are consistent with a previous study, which found that CCR5 expression on CD8^+^ T cells was increased in the TB, HIV, and HT group compared with the HC group, but there was no difference between the TB, HIV, and HT groups (25). In addition, DP, CD4^low^CD8^high^ and CD4^high^CD8^low^ T cells expressed higher CCR5 than CD4^+^ and CD8^+^ T cells in the HT group. This is likely a reflection of dynamic processes that DP T cells with initially CCR5 negative may have upregulated CCR5 expression due to immune activation associated with HIV and/or TB infection, thus becoming susceptible targets of infection. Significantly more total, CD4^low^CD8^high^ and CD4^high^CD8^low^ DP T cells than CD4^+^ or CD8^+^ T cells expressed the tissue-homing marker CXCR3, suggesting that these cells were more prone to migrate into tissues than CD4^+^ or CD8^+^ T cells.

Previous studies have demonstrated that the production of TNF-α by MTB-specific peripheral T cells was lower in HIV^+^ individuals than in HIV^−^ individuals with TB (26, 27). In our study, compared with the HC group, the percentage of DP and CD4^high^CD8^low^ T cells producing TNF-α was lower in the HIV and HT group, respectively. The percentage of DP and CD4^high^CD8^low^ T cells producing TNF-α was lower in the HT group than in the TB group. However, no differences in the percentage of DP T cells producing TNF-α were observed between the TB and HC groups nor between the HT and HIV groups. All these suggested that HIV infection, but not TB, might suppress TNF-α expression on DP T cells; or DP T cells expressing TNF-α might immigrate to the lesion location, leading to a lower level of TNF-α^+^ DP T cells in the peripheral blood. A previous study showed that HIV coinfection might impair IFN-γ and TNF-α secretion as well as the proliferative capacity of effector memory CD4^+^ T cells in TB (28). CD4^high^ CD8^low^ T cells, which were derived from late differentiated effector CD4^+^ T cells, might produce less level of TNF-α. Cytotoxic molecules, such as granzyme and perforin, are produced by cytotoxic lymphocytes, including cytotoxic T lymphocytes (CTLs) and natural killer (NK) cells. They contribute directly to immune defense against HIV infection and TB (29, 30). In our study, both HIV and HT groups had elevated granzyme A-producing capacity of total and CD4^low^CD8^high^ DP T cells. However, no differences in the percentage of DP T cells producing granzyme A were observed between the TB and HC groups. Therefore, HIV infection, but not TB, may enhance the ability of total and CD4^low^CD8^high^ DP T cells to increase apoptosis by increasing the secretion of granzyme A. Although granzyme A and perforin cooperate to induce target cell apoptosis (30), no differences in the percentage of perforin-producing DP T cells were observed in the HIV and HT group compared with the HC and TB group. In addition, we observed that DP T cells expressed more cytolytic markers (granzyme A and perforin) than CD4^+^ T cells, but not CD8^+^ T cells, and Th1 cytokines (IFN-γ and TNF-α) similar to CD4^+^ and CD8^+^ T cells in the HT group.

In conclusion, increased percentages of DP T cells were mainly derived from CD4^low^CD8^high^ T cells during HIV infection and HT coinfection, while they were mainly derived from both CD4^low^CD8^high^ and CD4^high^CD8^low^ T cells during TB. We speculated that increased DP T cells may be associated with the increased expression of CCR5, making DP T cells the site of pathogen replication. In addition, DP T cells had the same cytotoxic function as CD8^+^ T cells. Therefore, DP T cells may be an important part of cellular immunity in HIV and MTB infections, which contribute to the role of an immune protection.

## Data Availability Statement

The raw data supporting the conclusions of this article will be made available by the authors, without undue reservation.

## Ethics Statement

The studies involving human participants were reviewed and approved by the study was approved by the Research and Ethics Committee of Zhongnan Hospital, Wuhan University, P. R. China (Ethics, 2016009). Informed consent was obtained from all individuals enrolled in this study. The patients/participants provided their written informed consent to participate in this study.

## Author Contributions

LS and KL conceived and designed this investigation. SZ, YT, and YL helped design the scheme of the investigation and collected the original data. YX and QZ performed the experiments. WG, ML, and SW analyzed the data. SZ, YT, and KL contributed to the writing of this paper. All authors contributed to the article and approved the submitted version.

## Funding

This work was supported by the Medical Science and Technology Innovation Platform Support Project of Zhongnan Hospital, Wuhan University (PTXM2020008), the Science and Technology Innovation Cultivation Fund of Zhongnan Hospital, Wuhan University (cxpy2017043), Medical Science Advancement Program (Basic Medical Sciences) of Wuhan University (TFJC2018004), the Non-profit Central Research Institute Fund of Chinese Academy of Medical Sciences (2020-PT320-004), and Discipline Cultivation Project of Department of Infectious Diseases, Zhongnan Hospital, Wuhan University (ZNXKPY2021027).

## Conflict of Interest

The authors declare that the research was conducted in the absence of any commercial or financial relationships that could be construed as a potential conflict of interest.

## Publisher's Note

All claims expressed in this article are solely those of the authors and do not necessarily represent those of their affiliated organizations, or those of the publisher, the editors and the reviewers. Any product that may be evaluated in this article, or claim that may be made by its manufacturer, is not guaranteed or endorsed by the publisher.
