# Supplementary material for: The Role of CD4+CD8+ T Cells in HIV Infection With Tuberculosis
Source: Front Public Health. 2022 May 27;10:895179. doi: 10.3389/fpubh.2022.895179 (PMC9195591; doi:10.3389/fpubh.2022.895179)
Supplement: Supplementary file 1 [file Data_Sheet_1.DOCX]

**Figure legends:**

**Supplement Figure 1. Correlation between CD4 cell count and DP frequency among HIV and HT groups.** Multiple linear regression was employed to identify CD4 count associated with the DP frequency.

**Supplement Figure 2.** **Distribution of chemokines among subsets of DP T cells in each group.** Percentages of DP T subsets expressing CXCR4 (A) and CCR7 (B) are shown among each group. P value calculated using Mann–Whitney U test. Statistically signiﬁcant differences between the groups are indicated as follows: *P<0.05 and **P<0.01.

**Supplement Figure 3. Function of DP T cells among each group.** Percentages of DP T subsets producing IFN-γ (A) are shown among each group. Subjects producing cytokines of IFN-γ in T-lymphocyte subsets among HT group are shown in (D). P value calculated using Mann–Whitney U test. Statistically signiﬁcant differences between the groups are indicated as follows: *P<0.05 and **P<0.01.
