# Supplementary figures and images for: The Role of CD4+CD8+ T Cells in HIV Infection With Tuberculosis
Source: Front Public Health. 2022 May 27;10:895179. doi: 10.3389/fpubh.2022.895179 (PMC9195591; doi:10.3389/fpubh.2022.895179)

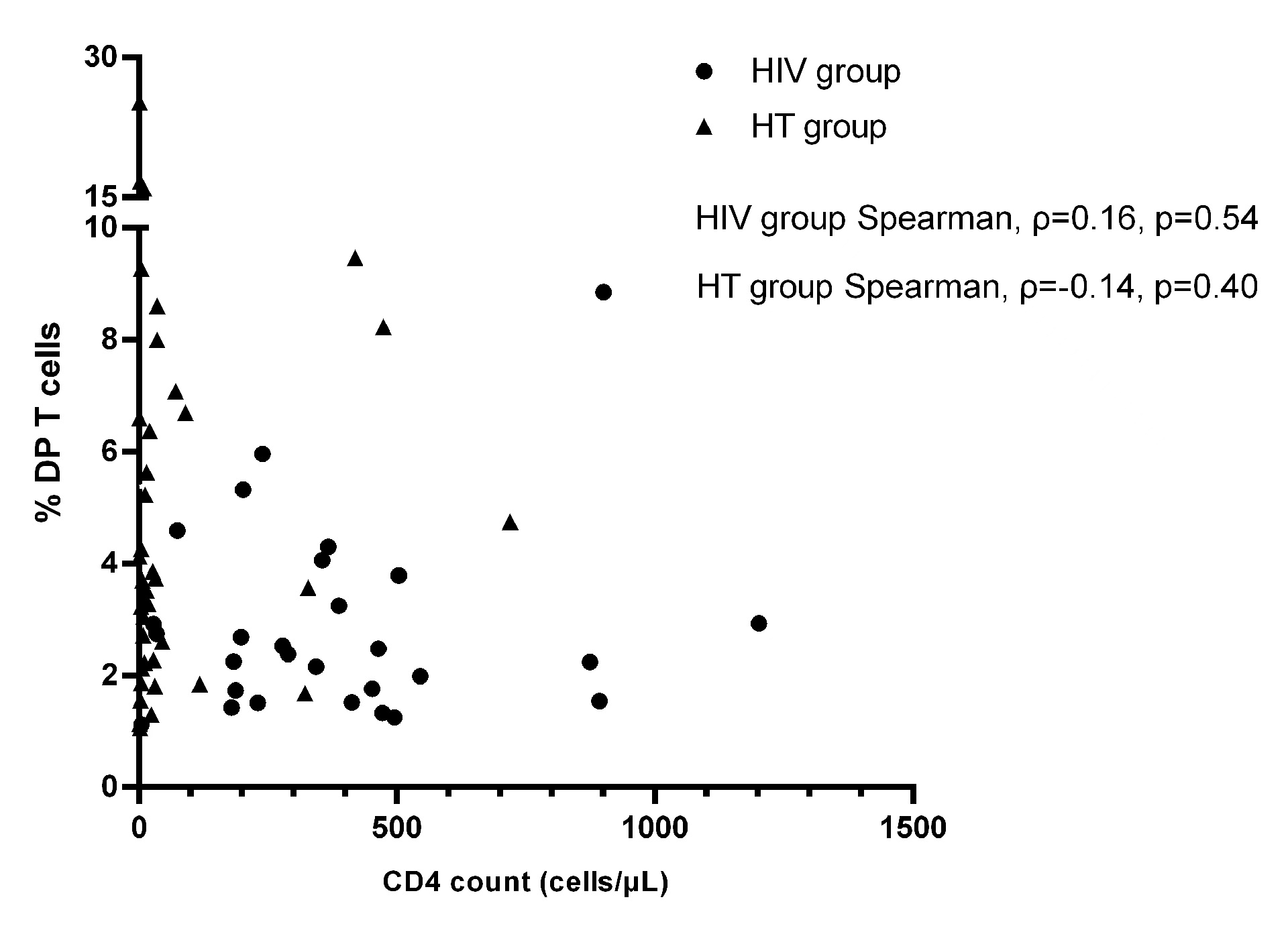

Supplement: Supplementary file 2 [file Image_1.JPEG]

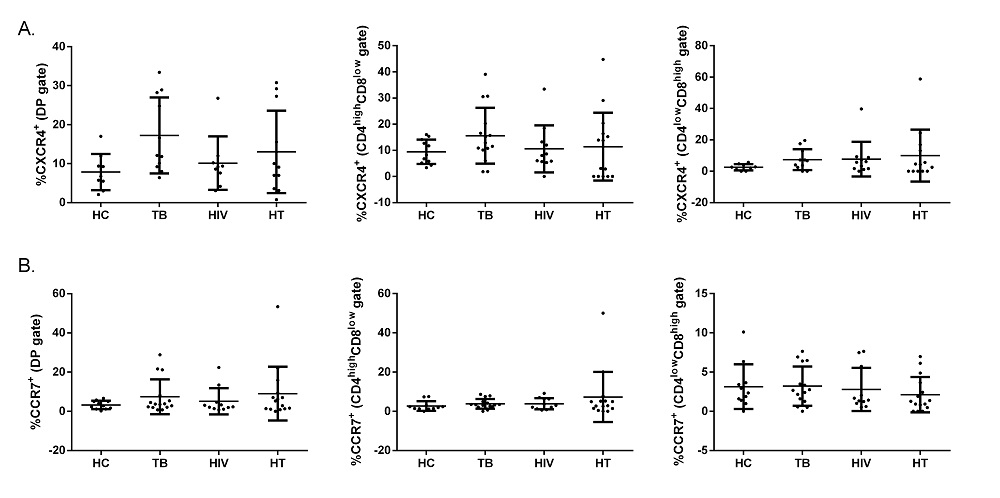

Supplement: Supplementary file 3 [file Image_2.JPEG]

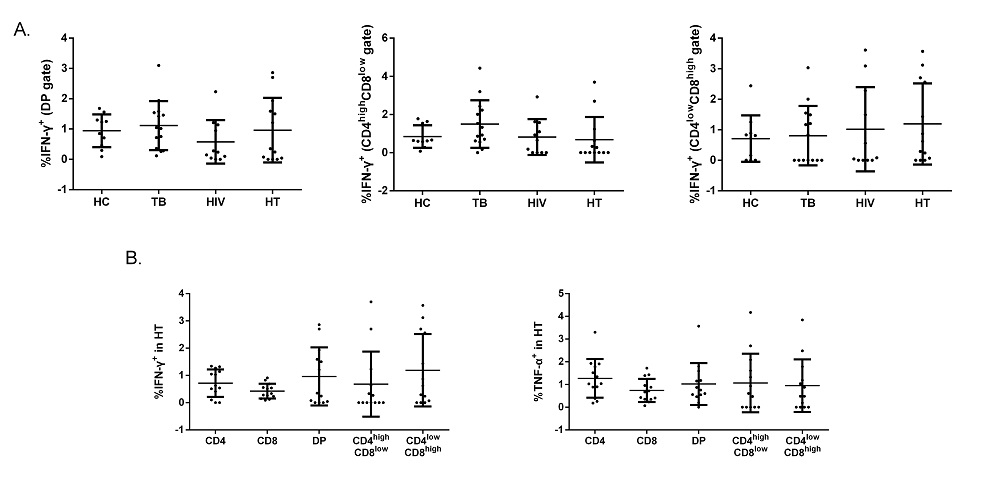

Supplement: Supplementary file 4 [file Image_3.JPEG]
